# Supplementary material for: Association between oral microbiome and seven types of cancers in East Asian population: a two-sample Mendelian randomization analysis
Source: Front Mol Biosci. 2023 Nov 21;10:1327893. doi: 10.3389/fmolb.2023.1327893 (PMC10702768; doi:10.3389/fmolb.2023.1327893)
Supplement: Supplementary file 1 [file DataSheet1.ZIP › Supplementary Materials/MR plots of saliva/saliva═╝/ovarian cancer/pheno.1099_to_ovarian cancer_forest.pdf]

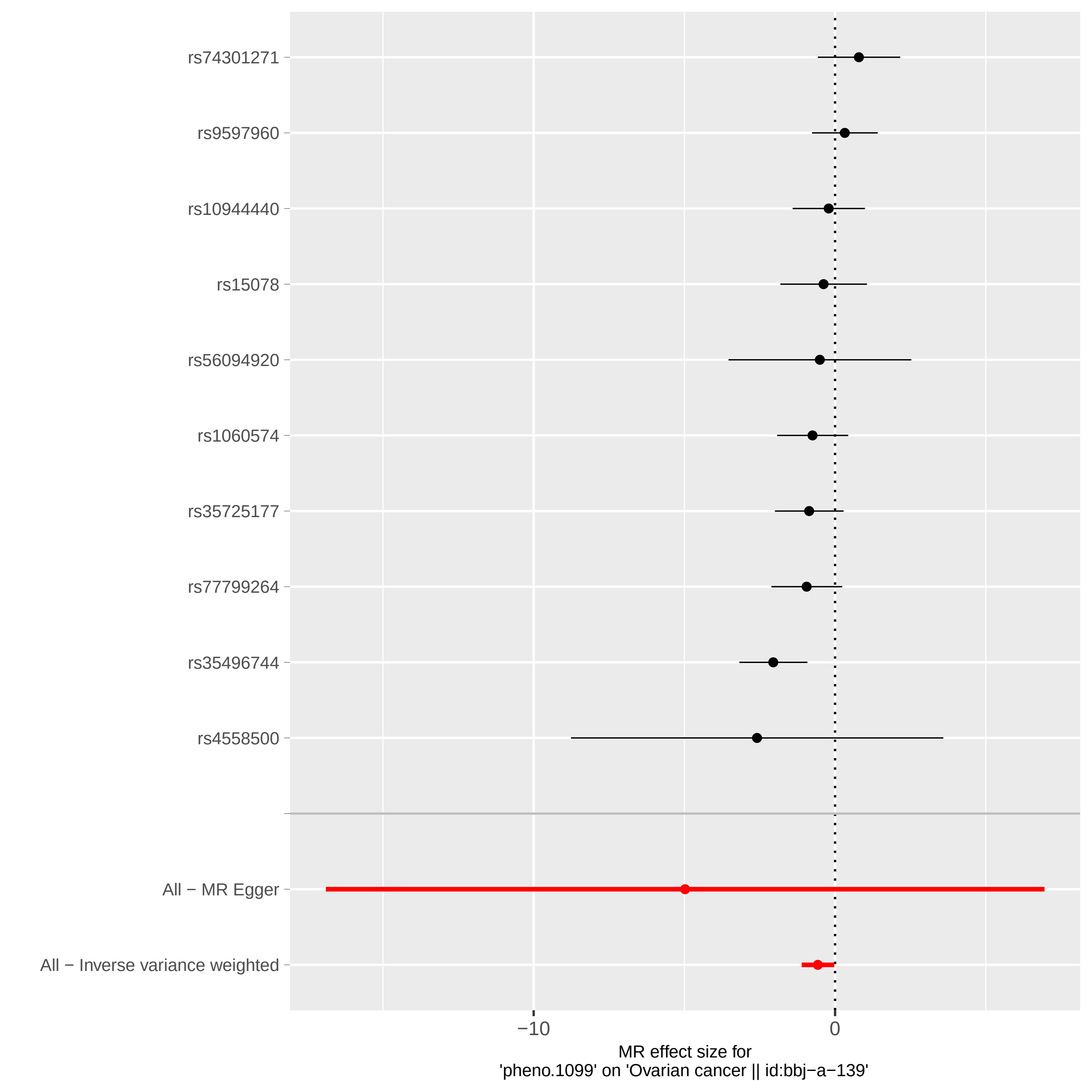
rs74301271
rs9597960
rs10944440
rs15078
rs56094920
rs1060574
rs35725177
rs77799264
rs35496744
rs4558500
All − MR Egger
All − Inverse variance weighted
−10
0
MR effect size for
'pheno.1099' on 'Ovarian cancer || id:bbj−a−139'
